# A Rare Report of Infectious Emphysematous Aortitis Secondary to* Clostridium septicum* without Prior Vascular Intervention

**DOI:** 10.1155/2017/4984325

**Published:** 2017-09-17

**Authors:** Ciel Harris, Joseph Geffen, Keyrillos Rizg, Stuart Shah, Aaron Richardson, Cherisse Baldeo, Avinash Ramdass

**Affiliations:** ^1^Internal Medicine, University of Florida College of Medicine-Jacksonville, Jacksonville, FL, USA; ^2^Pulmonary, Sleep and Critical Care Medicine, University of Florida College of Medicine-Jacksonville, Jacksonville, FL, USA

## Abstract

The term “mycotic aneurysm” was first used by Osler in 1882 to describe a mushroom-shaped aneurysm in subacute bacterial endocarditis. Mycotic aneurysms account for only 2.6% of all aneurysms of the aorta. Rarer still are anaerobic infections secondary to organisms such as* Clostridium septicum*, which results in emphysematous aortitis. The vast majority of emphysematous aortic infections occur as a result of instrumentation; however, in this case we present an infection de novo. A 75-year-old male presented with a 2-week history of progressive fatigue and chest pain that then developed into constitutional symptoms. Chest radiograph demonstrated an obvious widened mediastinum. CT angiogram of his chest then confirmed this finding as well as significant periaortic gas and focal outpouching. Numerous diverticuli with inflammatory changes consistent with diverticulitis was observed on CT abdomen. Blood cultures returned positive for* Clostridium septicum*. Definitive treatment was discussed including debridement and graft insertion; however, patient decided on conservative management and was discharged on intravenous antibiotics. Unfortunately, as in most cases of emphysematous aortitis that do not undergo surgical management, the patient succumbed to his illness. The lesson provided will be the epidemiology of emphysematous aortitis, presentation, diagnosis, management, and prognosis through a case report.

## 1. Introduction

In this case, we present an emphysematous arterial infection secondary to* Clostridium septicum* without the history of vascular intervention.

## 2. Case

**Figure 1:**
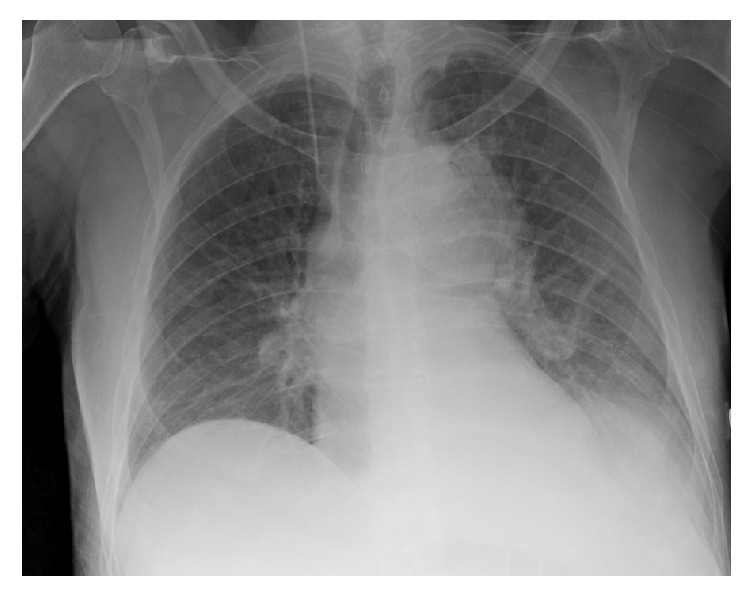
Chest radiograph showing widened mediastinum.

**Figure 2:**
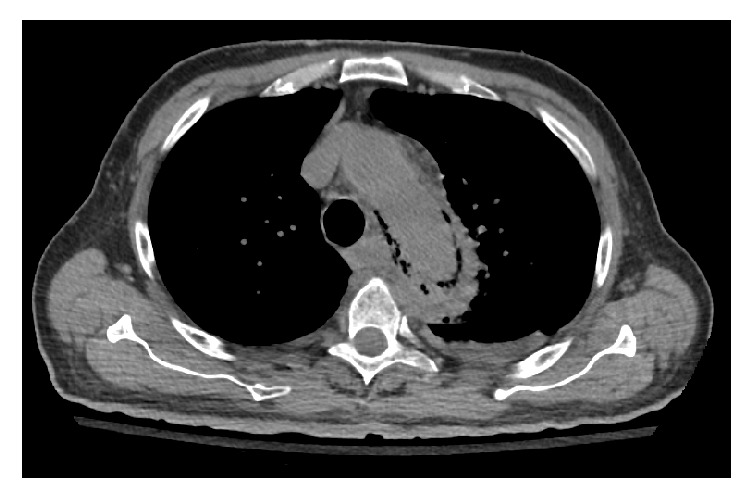
CT chest (sagittal view) showing emphysematous aortitis.

**Figure 3:**
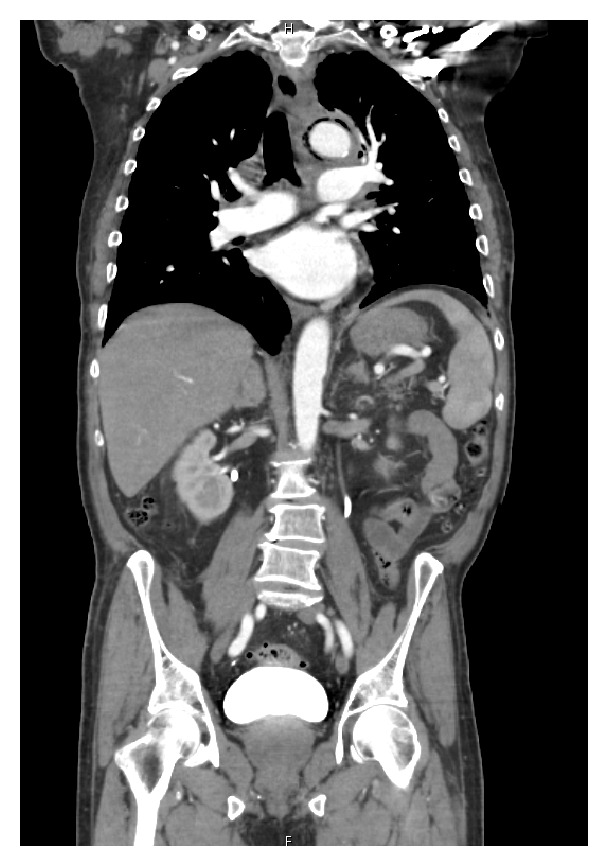
CT chest (coronal view) showing emphysematous aortitis.

**Figure 4:**
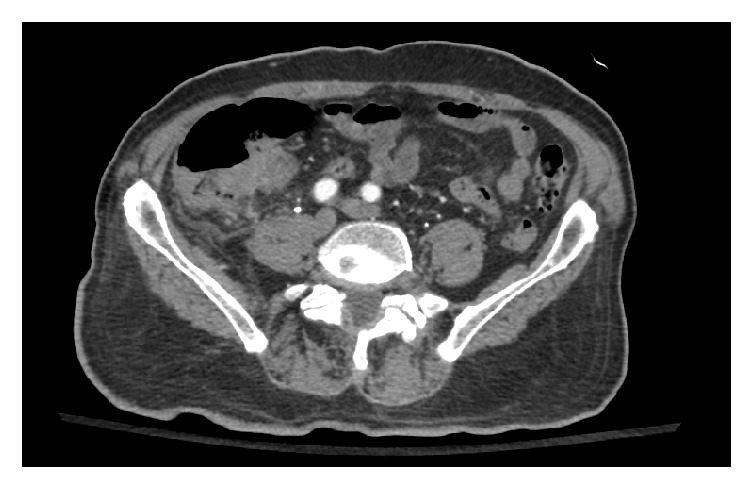
CT abdomen showing evidence of cecal diverticulitis.

A 75-year-old male with a past medical history of hypertension and diabetes mellitus presented to our outpatient clinic with a 3-week history of progressive fatigue associated with intermittent left sided sharp chest pain that radiated to the mid-axilla bilaterally. He denied any exertional symptoms but endorsed subjective fevers and chills of two-day duration. Important negatives included dyspnea, lightheadedness, palpitations, syncope, and no gastrointestinal symptoms. Examination findings were all within normal limits including vital signs and cardiovascular exam. Lab investigations were significant for a leukocytosis with an associated left shift. Chest X-ray (Figure 1) showed a widened mediastinum. Chest CT angiogram demonstrated thoracic aortic wall thickening with significant periaortic gas and small focal outpouching concerning for mycotic aneurysm (Figures 2 and 3). CT abdomen also demonstrated numerous colonic diverticuli with inflammatory changes in the cecum consistent with diverticulitis (Figure 4). He was started on broad spectrum antibiotics and esmolol for heart rate control. Blood cultures revealed Gram-positive rods consistent with* Clostridium septicum* (CS) susceptible to piperacillin-tazobactam. Cardiothoracic surgery was consulted for definitive surgical management entailing a left thoracotomy and distal aortic arch replacement with a Dacron (PET, polyethylene terephthalate); however, the patient declined surgical intervention. Antibiotics were continued for conservative management and sterilization of the aorta for 3 weeks with resolving bacteremia on repeat blood cultures. Unfortunately, during this time, the patient expired.

## 3. Discussion

Emphysematous aortitis (EA) is a rare but serious condition associated with significant morbidity and mortality [1–5]. EA occurs when there is an underlying mycotic aneurysm secondary to a gas producing organism. Most reported cases of emphysematous aortitis are due to endovascular graft complication; here we present an infection either de Novo or more likely from concurrent diverticulitis. Mycotic aneurysms occur via four mechanisms: direct bacterial inoculation (trauma), bacteremic seeding (as in this case), contiguous infection, or septic emboli [1]. Identifiable risk factors for mycotic aneurysm in this patient include impaired immune system from long standing diabetes as well as atherosclerotic plaques. The most common organisms responsible for mycotic aneurysms are staphylococcus and salmonella which are not gas producing. CS is a Gram-positive obligate anaerobic organism that is a part of normal gut flora. CS causes infection through myonecrosis and release of exotoxins. To our knowledge, there are only 16 cases of EA due to CS published until 2001, with a current literature review revealing 2 cases, neither of which was reported in the United States [2, 3]. While CS infection is well reported to be associated with traumatic injury, there is also an established relationship with malignancy, both hematologic and gastrointestinal [6]. Treatment of CS infection includes immediate institution of antibiotics and surgical debridement. The first-line antibiotic is penicillin [1, 5] with second line (reserved for penicillin-allergies) including 3rd- and 4th-generation cephalosporins, metronidazole, imipenem, or vancomycin. It is recommended that these agents are combined with others for broad spectrum coverage. Definitive treatment for this patient would have been immediate debridement of infected aorta with prolonged antibiotic therapy of at least 6–8 weeks after procedure. Prognosis is poor without intervention with a staggering mortality rate of at least 50–100% [1–3]. Death of this patient was presumed to be due to aortic aneurysm rupture versus septicemia. If he did survive with antibiotic management alone or had proceeded to surgery, we would have recommended aggressive malignancy workup.

## References

[1] Spelmen D. (2016). Overview of infected (mycotic) arterial aneurysm. *UpToDate*.

[2] Morrison R. (2016). Clostridial mycotic aneursym of the thoracoabdominal aorta, a case report. *Vascular Surgery*.

[3] Seder C. W., Kramer M., Long G., Uzieblo M. R., Shanley C. J., Bove P. (2009). Clostridium septicum aortitis: report of two cases and review of the literature. *Journal of Vascular Surgery*.

[4] Koransky J. R., Stargel M. D., Dowell V. (1979). Clostridium septicum bacteremia and its clinical significance. *The American Journal of Medicine*.

[5] Granier M., Granier A., Fraga J., Durant R. (2011). Emphysematous infectious aortitis: a dramatic evolution. *European Heart Journal*.

[6] Chew S. S. B., Lubowski D. Z. (2001). Clostridium septicum and malignancy. *ANZ Journal of Surgery*.

